# Serotonergic mediation of the brain-wide neurogenesis: Region-dependent and receptor-type specific roles on neurogenic cellular transformation

**DOI:** 10.1016/j.crneur.2023.100102

**Published:** 2023-07-28

**Authors:** Yuki Higuchi, Hiroyuki Arakawa

**Affiliations:** Department of Systems Physiology, Graduate School of Medicine, University of the Ryukyus, Okinawa, Japan

**Keywords:** Serotonin (5-HT), Neurogenesis, 5-HT receptors, Neural stem cells, Antidepressant

## Abstract

Brain serotonin (5-hydroxytryptamine, 5-HT) is a key molecule for the mediation of depression-related brain states, but the neural mechanisms underlying 5-HT mediation need further investigation. A possible mechanism of the therapeutic antidepressant effects is neurogenic cell production, as stimulated by 5-HT signaling. Neurogenesis, the proliferation of neural stem cells (NSCs), and cell differentiation and maturation occur across brain regions, particularly the hippocampal dentate gyrus and the subventricular zone, throughout one's lifespan. 5-HT plays a major role in the mediation of neurogenic processes, which in turn leads to the therapeutic effect on depression-related states. In this review article, we aim to identify how the neuronal 5-HT system mediates the process of neurogenesis, including cell proliferation, cell-type differentiation and maturation. First, we will provide an overview of the neurogenic cell transformation that occurs in brain regions containing or lacking NSCs. Second, we will review brain region-specific mechanisms of 5-HT-mediated neurogenesis by comparing regions localized to NSCs, i.e., the hippocampus and subventricular zone, with those not containing NSCs. Highlighting these 5-HT mechanisms that mediate neurogenic cell production processes in a brain-region-specific manner would provide unique insights into the role of 5-HT in neurogenesis and its associated effects on depression.

## Highlights

• 5-HT mediates neurogenic process as a major player in antidepressant mechanism.
• Neurogenesis can be observed in brain-wide regions with or without neural stem cells.
• 5-HT receptor subtypes act as phase mediators across neurogenic cellular transformations.
• 5-HT receptors also contribute to neurogenic processes in non-neural stem cell brain locations.

## Graphical abstract

**Figure:**
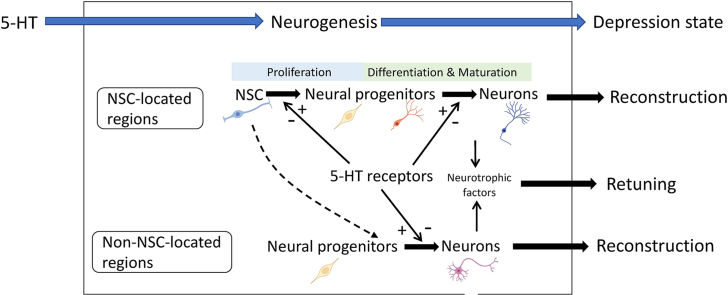

## Abbreviations

BDNF: Brain-derived neurotrophic factor
BrdU: Bromodeoxyuridine
CA: Cornu Ammonis
CNS: Central nervous system
CREB: cAMP response element-binding protein
DCX: Doublecortin
DHT: 5,7-dihydroxytryptamine
DG: Dentate gyrus
DRN: Dorsal raphe nucleus
GCL: Granule cell layer
GFAP: Glial fibrillary acidic protein
LV: Lateral ventricle
ML: Molecular layer
NSC: neural stem cell
OB: Olfactory bulb
PCPA: para-chlorophenylalanine
RMS: Rostral migratory stream
SGZ: Subgranular zone
SSRIs: Selective serotonin reuptake inhibitors
SVZ: Subventricular zone
5-HT: 5-Hydroxytryptamine (serotonin)

## 1 Antidepressant effects of 5-HT via mediation of neurogenesis

For several decades, humans with major depressive disorder and other mental and neurological disorders (Moreno-Agostino et al., 2021; Smith, 2014) have been using therapeutic antidepressant treatment against depression-induced imbalanced states with neurotransmitters. Most antidepressants are involved in the modification of serotonin (5-hydroxytryptamine, 5-HT) action in the brain (Pereira and Hiroaki-Sato, 2018). The enhancement of 5-HT transmission leads to the retuning of the neural signal, orchestrated with other neurotransmitters like norepinephrine and dopamine, that govern behavioral outputs that exhibit depression (El Mansari et al., 2010). Selective serotonin reuptake inhibitors (SSRIs), the most commonly prescribed antidepressant initially decreases the firing rate of 5-HT neurons, and its repeated ingestion thereupon results in the discharge of the firing, increasing of 5-HT levels in synaptic clefts due to desensitization of the 5-HT autoreceptor, and the inhibition of the reuptake processes (Blier and Ward, 2003; Haleem, 2019). Monoamine oxidase inhibitors, another type of antidepressant, also increase 5-HT levels by preventing the degradation of released 5-HT in the synaptic clefts (Higuchi et al., 2017).

The mode of action of antidepressants to modulate the depression-like state by influencing the 5-HT transmissions is still unclear. A growing body of evidence indicates neurogenic processes in the central nervous system (CNS) are responsible for the route of antidepressant efficacy against depressive behavior (Hanson et al., 2011; Mahar et al., 2014). Accordingly, one argument for the need to increase neuronal generation in the brain by antidepressants is that the abnormal behaviors and negative experiences responsible for poor mental health also influence neurogenesis in adults (Samuels et al., 2011, Schoenfeld and Cameron, 2015). Depression is also characterized by the disruption of neural circuitry and neurogenesis (Berger et al., 2020). Neurogenesis, the process of neuronal cell production, is preserved from the prenatal stage to adulthood, wherein new neurons are generated and integrated in the brain (Moreno-Jiménez et al., 2019), and it entails the regrowth of lost connections to reform the neuronal networks (Denoth-Lippuner and Jessberger, 2021). 5-HT contributes to this process as one of the crucial signals in the process of neurogenesis, along with other neurotransmitters and growth factors responsible for regulating cell proliferation and differentiation (Eliwa et al., 2017). The neurogenic process occur in a few spatially-restricted brain regions due to the discrete existence of neural stem cells (NSCs) in the mammalian brain (Taupin, 2006); these two regions include the subventricular zone (SVZ) located along the lateral ventricle (Gheusi et al., 2000) of the brain, and the subgranular zone (SGZ) of the dentate gyrus (DG) of the hippocampus (Akers et al., 2014).

**Fig. 1:**
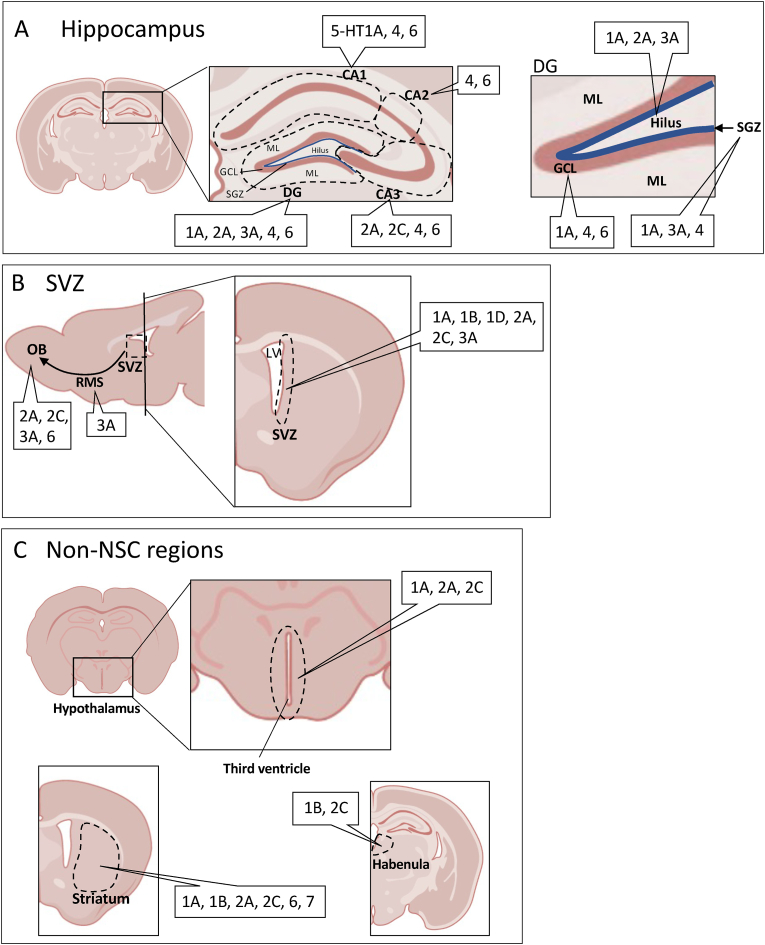
Localization of different subtypes of 5-HT receptors in the hippocampus, SVZ and non-NSC-located regions. (A) In the hippocampus, different sets of 5-HT receptor subtypes are expressed in the different subregions of the hippocampus. Neural stem cells and neural progenitor cells are localized in the SGZ of the DG, while mature newborn neurons localize in the GCL of the DG. (B) In the SVZ, 5-HT1A, 1B, 1D, 2A, 2C, 3A receptors are expressed. Among these subtypes, 5-HT3A receptors are expressed in the neuroblasts along the RMS (indicated as a curved arrow between the SVZ and OB). (C) Non-NSC-located regions, including the hypothalamus, striatum and habenula also express several subtypes of 5-HT receptors. *CA, cornu Ammonis; DG, dentate gyrus; GCL, granule cell layer; LV, lateral ventricle; ML, molecular layer; NSC, neural stem cell; OB, olfactory bulb; RMS, rostral migratory stream; SGZ, subgranular zone; SVZ, subventricular zone; 5-HT, 5-hydroxytryptamine (serotonin).*

While 5-HT plays a pivotal role in these typical neurogenic niches, the mechanisms by which 5-HT acts brain-wide to mediate the spatiotemporal processes of neurogenesis across various brain regions remain undetermined. Several studies have demonstrated that neurogenic neuronal integration by 5-HT stimulation is observed in regions where NSCs do not reside (Feliciano et al., 2015; Jurkowski et al., 2020; Kempermann et al., 2015; Lim and Alvarez-Buylla, 2016; Morales and Mira, 2019), suggesting that the 5-HT-mediated regulation of neurogenic processes may not be limited to the regions containing NSCs alone (Fig. 1). Therefore, this review aims to illustrate the orchestrated mediation of 5-HT signals in the brain-wide neurogenic process, mainly based on the findings from rodent model studies. We will review recently published literature for a better understanding of the circuit mechanisms of 5-HT in the mediation of region-typical neurogenesis and its further downstream pathways responsible for the depression-like state.

## 2 Region and cell-typical processes of neurogenesis

### 2.1 Neural stem cells-derived processes of neurogenesis

Neurogenesis is derived from the hatching of NSCs that reside in the CNS of mammals (Taupin and Gage, 2002). The cellular transformation of neurogenesis occurs through 1) cell proliferation and 2) cell-type differentiation and neuronal maturation (Niklison-Chirou et al., 2020). In the proliferation phase, neurogenesis occurs to hatch the NSCs that retain cell fate plasticity in restricted brain regions, including the DG and SVZ (Fuentealba et al., 2012; Morales and Mira, 2019). NSCs can be detected by the coexpression of nestin (a neural stem cell marker) and glial fibrillary acidic protein (GFAP, an astrocytic marker) (Filippov et al., 2003) (see Table 1). Subsequently, NSCs give rise to neural progenitor cells (NPCs), which are characterized as the neural cell-generating cell types, i.e., the neuronal and glial cells, but not as nonneural cells, such as the immune cells (Martínez-Cerdeño and Noctor, 2018). NPCs can be labeled with bromodeoxyuridine (BrdU), a synthetic nucleoside analog, as it gets incorporated with the newly generated DNA of these proliferating cells (Taupin, 2007).

**Table 1:** Cell type classification in the neurogenesis in the hippocampus, SVZ, and non-NSC regions, and the markers for identifying the cell type.

.Phase	Localization	Cell type	Markers for cell type identification						References
***Hippocampus***			Nestin	GFAP	DCX	CalR	CalB	NeuN	
Proliferation	SGZ	Type-1 stem cells	+	+					Filippov et al. (2003)
Proliferation	SGZ	Type-2A cells	+	-					(Filippov et al., 2003; Kronenberg et al., 2003)
Proliferation	SGZ	Type-2B cells	+	-	+				(Kronenberg et al., 2003; Nicola et al., 2015)
Differentiation									
Proliferation	SGZ	Type-3 cells (neuroblasts)	-	-	+				Kronenberg et al. (2003)
Differentiation									
Maturation	GCL	Immature neurons			+	+	-	±	Brandt et al. (2003)
Maturation	GCL	Mature neurons			-	-	+	+	Ambrogini et al. (2004)
***SVZ-OB stream***			Nestin	GFAP	DCX	CalR	CalB	NeuN	
Proliferation	SVZ	Type-B (RGL) stem cells	+	+					(Doetsch et al., 1997, 1999; Mirzadeh et al., 2008)
Proliferation	SVZ	Type-C cells	+	-	-				(Doetsch et al., 1997; Kim et al., 2009; Liu and Crews, 2017)
Proliferation	SVZ-OB (migrating)	Type-A cells (neuroblasts)	-	-	+	-	-	-	(Doetsch et al., 1997; Kim et al., 2009; Liu and Crews, 2017; Lois et al., 1996)
Differentiation									
Maturation	OB	Mature neurons	-	-	-	±*	±*	+	(Merkle et al., 2007; Young et al., 2007)
**Non-NSC regions**			Nestin	GFAP	DCX			NeuN	
?	Hypothalamus	α2-Tanycytes	+	+					(Robins et al., 2013; Sachs and Caron, 2014)
		β1-Tanycytes	+	-					
?	Habenula		+						Sachs and Caron (2014)
?	Striatum (ischemic)	SVZ-derived cells	+						Jin et al. (2003)
?		RMS-derived cells			+				Jin et al. (2003)
?	Cortex (ischemic)	Unknown cells	+	+					Jin et al. (2003)
?		Unknown cells			+			+	Jin et al. (2003)

CalB, Calbindin; CalR, calretinin; DCX, doublecortin; DG, dentate gyrus; GCL, granular cell layer; GFAP, Glial fibrillary acidic protein; OB, olfactory bulb; RGL, radial-glia-like; SGZ, subgranular zone; SVZ, subventricular zone. *Mature adult-born neurons derived from the SVZ are categorized into CalR-positive or CalB-positive cells.

The expression of BrdU-labeled cells has been reported in non-NPCs located in regions such as the cortex (Kodama et al., 2004; Soumier et al., 2010), striatum (Soumier et al., 2010), hypothalamus (Ohira, 2022; Sachs and Caron, 2014), and habenula (Sachs and Caron, 2014). One possible mechanism is that hatching NSCs or proliferating NPCs might migrate from their origin regions (i.e., SVZ and SGZ), and indeed, NPCs derived from the SVZ migrate to the olfactory bulb (OB) via the rostral migratory stream (RMS), a route for neuroblasts via the OB that takes place throughout life (James et al., 2011). Contrarily, it is argued that BrdU labeling occurs even in nonproliferating cells or that unknown processes are involved in BrdU incorporation.BrdU can be incorporated into genomic DNA not only during cell proliferation, but also during DNA repair (Zheng et al., 2011). BrdU incorporation seems to occur when the rate of DNA repair is accelerated following irradiation-induced DNA damage (Beisker and Hittelman, 1988; Selden et al., 1993, 1994). To this extent, neurogenic processes represented by BrdU labeling can be observed in several non-NPC-located brain regions following electrolytic injury (Cao et al., 2002), ischemia (Magavi et al., 2000; Osman et al., 2011), or high-fat diet exposure (Gouazé et al., 2013). Such technical limitations to the detection of proliferating cells must be considered for the characterization of the region-specific cell transformation process of neurogenesis.

In subsequent cell-type differentiation and maturation phases, the proliferating cells are further converted to transiently amplifying progenitor cells, which is detectable by the transient expression of the neuronal differentiation marker, doublecortin (DCX) (Kempermann et al., 2015). These cell types are characterized as nestin-positive but GFAP-negative, unlike NSCs (Kempermann et al., 2004). These differentiated neuronal cells are integrated with preexisting cells into mature cells, accompanied by a decline in DCX expression (Brown et al., 2003), and the maturation process is mediated by the expression of neurotrophic factors, such as brain-derived neurotrophic factor (BDNF) (Ortiz-López et al., 2017). The DCX-positive cells are detected throughout the brain (Jurkowski et al., 2020), along with BDNF levels (Vilar and Mira, 2016). These neuroblast populations, which are accompanied by neurotrophic factors, play a significant role in neuronal repair and formation (Wu et al., 2023). We argue a possible mechanism that these neuroblast cell processes in regions not containing NSCs occur independently of the neurogenic processes derived from hatching NSCs and that 5-HT signaling may mediate these discrete neurogenic processes across regions. To address this, the region-specific processes of neurogenesis with NSCs-dependent and independent pathways have been summarized in Table 1.

### 2.2 Region-specific process of neurogenesis

**Fig. 2:**
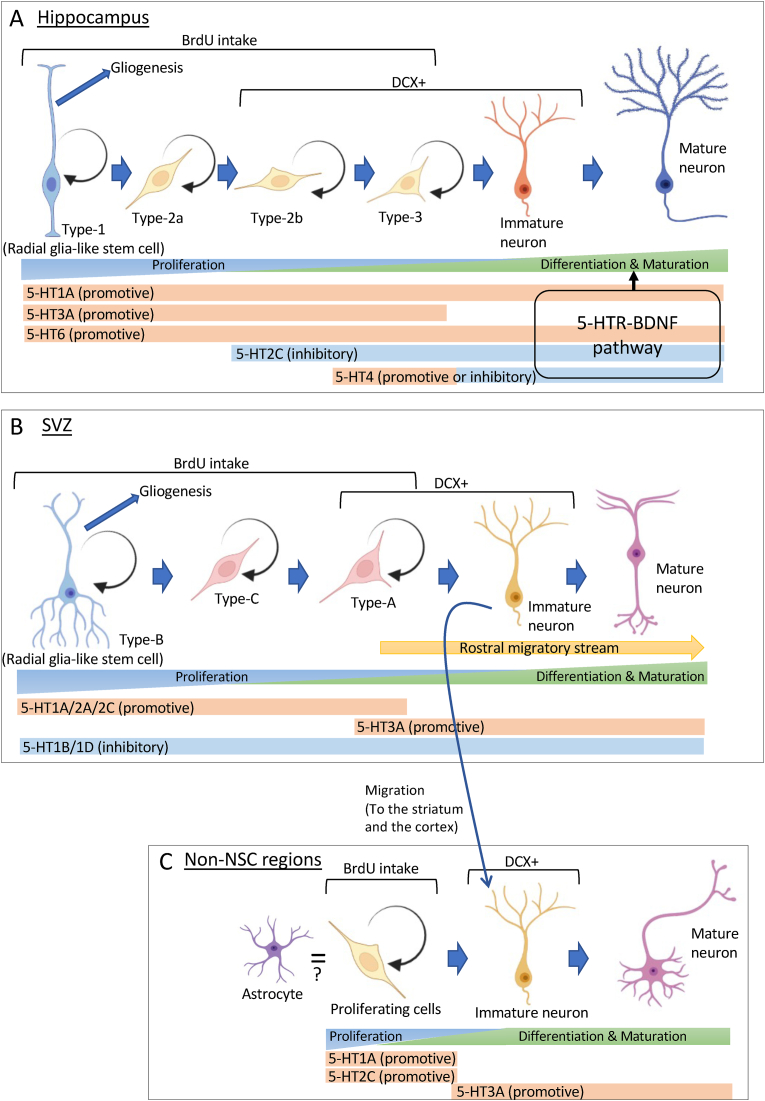
Mediatory mechanism of neurogenesis by differential 5-HT receptor subtypes in the hippocampus, SVZ, and non-NSC regions. (A) Cell proliferation promoted by 5-HT1A/3A/4/6 receptors but inhibited by 5-HT2C receptors in the hippocampus. Differentiation and maturation mediated by 5-HT1A/2C/4/6 receptors. Effects of 5-HT1A/2C/6 receptors on differentiation and maturation mediated through BDNF expression. (B) Proliferation promoted by 5-HT1A/2A/2C receptors but inhibited by 5-HT1B/1D receptors in the SVZ. Differentiation and maturation activated by 5-HT3A receptors. (C) In the non-NSC regions, such as the striatum, cortex, and hypothalamus, 5-HT1A/2C receptors promote cell proliferation and 5-HT3A receptors promote neuronal differentiation and maturation. A part of DCX-positive cells in the striatum and the cortex derived from the SVZ (Dayer et al., 2005; Shapiro et al., 2007; Yamashita et al., 2006). *BDNF, brain-derived neurotrophic factor; BrdU, Bromodeoxyuridine; DCX, doublecortin; SVZ, subventricular zone; 5-HT, 5-hydroxytryptamine.*

The SGZ of the DG in the hippocampus is one of the major sources of NSCs in the mammalian brain (Spalding et al., 2013) (Fig. 1A). In the DG, NSCs are called Type-1 cells (Kempermann et al., 2004) and NPCs are referred to as Type-2 and Type-3 cells (Li and Guo, 2020), which are not ambulant outside of the hippocampus but stably proliferating and integrating into mature cells within the hippocampal cell population (Toni and Schinder, 2015) (Fig. 2A). In cell-type differentiation and maturation phases, amplifying progenitor cells, referred to as Type-2A and 2B cells, remain in the DG (Brown et al., 2003; Filippov et al., 2003; Kempermann et al., 2015; Kronenberg et al., 2003). The DG Type-2A cells are DCX-positive, whereas Type-2B cells are DCX-negative (Brown et al., 2003; Kronenberg et al., 2003). Cell-type differentiation occurs following the emergence of a Na + current (Filippov et al., 2003), and Type-2B cells then give rise to Type-3 cells (Brandt et al., 2003). Type-3 cells are characterized as nestin-negative and DCX-positive, and they integrate with preexisting cells into mature cells, accompanied by a decline in DCX expression (Brown et al., 2003). Finally, Type-3 cells transport to the granular cell layer (GCL) of the hippocampus to give rise to immature neurons and are integrated into the neuronal network. Thus, NSC-derived cells play a predominant role in the neurogenic processes in the hippocampus and seem to contribute to entire processes within the hippocampal cell population.

The SVZ lining the lateral ventricles is one of the two largest neurogenic areas in the adult mammalian brain (Doetsch et al., 1999) (Fig. 1B). One typical characteristic of the SVZ region in contrast to the neurogenic region of the hippocampus is that immature neurogenic cells derived from the NSCs, called Type-B cells (Doetsch et al., 1997), migrate anteriorly along the RMS to their destination in the OB (Carleton et al., 2003, Gheusi et al., 2000; Lim and Alvarez-Buylla, 2016). Thereupon, NSCs generate rapidly dividing transit-amplifying progenitor cells, which differentiate into Type-C cells, which divide into Type-A cells (Lacar et al., 2010, Li and Guo, 2020), giving rise to postmitotic neuroblasts during the migration (Doetsch et al., 1999) (Fig. 2B). Type-C cells actively proliferate, are nestin-positive, GFAP-negative, and DCX-negative, and are similar to Type-2A cells in the DG (Brill et al., 2009; Ming and Song, 2011). Type-A cells are DCX-positive, unlike Type-C cells, migrating neuroblasts that move into the OB through an astrocytic tunnel-like structure ending in the OB (Sawada and Sawamoto, 2020). In the OB, migrating Type-A cells transform into granule cells (>95%) and periglomerular cells (<5%) (Lois and Alvarez-Buylla, 1994; Winner et al., 2002) and are integrated into existing neuronal circuitry, such as GABAergic interneurons (Lois and Alvarez-Buylla, 1994), to process odor information (Carlén et al., 2002; Kedrov et al., 2019). Across these differentiation and maturation phases, immature Type-C and Type-A cells receive support from BDNF to achieve maturation (Kirschenbaum and Goldman, 1995; Pencea et al., 2001). Although following an individual cell during their entire trajectories is not possible thus far, NSCs-derived cells in the SVZ seem to be migrated and integrated mainly into specific cell populations in the granular and periglomerular layers of the OB and take up specific functions for the olfactory process.

As for the brain regions where NSCs do not exist, DCX-positive cells are still observable in the brain regions, including the striatum (Ernst et al., 2014; Lv et al., 2018), hypothalamus (Batailler et al., 2014),cortex (Klempin et al., 2011), and amygdala (Jhaveri et al., 2018) (Fig. 1C). DCX is essential for neuronal migration and thus decreases its expression in immature neurons and disappears in mature neurons (Brown et al., 2003; Filippov et al., 2003; Kempermann et al., 2015; Kronenberg et al., 2003, Ponti et al., 2013). Therefore, an unexpected detection of DCX-positive cells implies that immature cells persist to exist in the targeted brain regions, although some subsets of DCX-positive cells may be from the SVZ in origin, arising via migration through the RMS (Dayer et al., 2005; Kreuzberg et al., 2010) (Fig. 2B and C). This suggests that certain neurogenic processes independent of the NSCs-derived cell population occur in several brain regions, and cell migration from the hippocampus and the SVZ is not a major source for the neurogenic process in the regions containing NSCs. Neurogenic processes, including cell proliferation, differentiation, migration, and maturation, are executed in a cell-type- and region-specific manner. This orchestration of cell development should be spearheaded by neurotrophic factors that locally mediate each process of neurogenesis. In the subsequent chapter, we will demonstrate how 5-HT-receptor systems are cardinal in the phasic regulation of region-specific processes of neurogenesis as neurotrophic factors.

## 3 5-HTmediation in the processes of neurogenesis

### 3.1 How does 5-HT act to mediate neurogenesis?

The dorsal raphe nucleus (DRN) in the midbrain is the major origin of central 5-HT, providing widespread 5-HT innervations to the forebrain via axonal transportation. Accordingly, approximately 50–60% of the 5-HT neurons in the rat and human brains are localized to the DRN (Lawther et al., 2020). Brain 5-HT neurons are widespread in the brain, reaching most cortical regions and noncortical ganglia (Lawther et al., 2020), including the cortex, hippocampus, amygdala, and hypothalamus (Bang et al., 2012; Hensler, 2006; Lesch and Waider, 2012; Mongeau et al., 1997). The hippocampus and the SVZ, both of which harbor the majority of NSCs, also receive 5-HT projections from the DRN and express 5-HT receptors (Chen et al., 2012; Dale et al., 2016; Fomin-Thunemann and Garaschuk, 2022) (Fig. 1A).

In the projecting sites, 5-HT mediates the biological processes of neurogenesis via synaptic binding to postsynaptic 5-HT receptors (Noto et al., 2016; Yohn et al., 2017), and several reports have indicated direct mediation by 5-HT in neurogenic cell production (David et al., 2009; Klempin et al., 2010; Kodama et al., 2004; Ohira, 2022; Sachs and Caron, 2014; Wang et al., 2008; Zavvari et al., 2020). A depletion of 5-HT synthesis by injection of 5-HT-specific neurotoxins (5,7-dihydroxytryptamine [DHT]) or a 5-HT-synthesizing enzyme inhibitor (para-chlorophenylalanine [PCPA]) leads to a reduced production of BrdU-positive neurogenic cells in both the hippocampal DG and SVZ (Brezun and Daszuta, 1999). Similarly, the efficacy of 5-HT enhancement on the production of hippocampal neurogenesis has also been documented following chronic treatment with SSRIs (David et al., 2009; Klempin et al., 2010; Wang et al., 2008; Zavvari et al., 2020). Treatments with SSRIs are of particular interest owing to the neurogenic effects evident in the nonhippocampal regions, such as the prefrontal cortex (Kodama et al., 2004) and hypothalamus (Ohira, 2022; Sachs and Caron, 2014).

The abundant effects of 5-HT on neurogenic processes are achieved by the subtype-specificity of 5-HT receptors. Cell proliferation in both DG and SVZ is promoted by 5-HT1A receptors (Banasr et al., 2004; Bert et al., 2006; Grabiec et al., 2009; Günther et al., 2011; Klempin et al., 2010; Noto et al., 2016; Radley and Jacobs, 2002; Soumier et al., 2010), in contrast to 5-HT2C receptors, which are involved in an inhibitory process of cell proliferation in these regions (Klempin et al., 2010; Soumier et al., 2009). Furthermore, 5-HT3 (Kondo et al., 2018), 5-HT4 (Mendez-David et al., 2014), and 5-HT6 (de Foubert et al., 2007, 2013) receptors are also implicated in the regulation of neurogenesis in the different phases. Such region-specific distributions of 5-HT receptors and a variety of these actions on neurogenic processes indicate that the 5-HT receptor subtypes may act on mediating the phases of the neurogenic processes in a brain-region-specific fashion, including the regions not containing NSCs or NPCs.

### 3.2 5-HT-mediated hippocampal neurogenic processes

The neurogenic cells, including NSCs and NPCs, are located at the SGZ of the hippocampal DG of the rodent brain, and they increase the axonal output to CA3 regions in the maturation phase (Faulkner et al., 2008; Gu et al., 2012). 5-HT signals are involved in the neurogenic processes in these regions via several subtypes of receptors, including 5-HT1A (Klempin et al., 2010), 5-HT2A (Banasr et al., 2004), 5-HT2C (Klempin et al., 2010), 5-HT3 (Kondo et al., 2018), 5-HT4 (Mendez-David et al., 2014), and 5-HT6 (de Foubert et al., 2013) (Fig. 1A). Among these 5-HT receptor subtypes, most (i.e., 5-HT1A, 2A, 3, 4, and 6) are expressed in the DG subregion of the hippocampus, where most of the NSCs are localized, whereas 5-HT2C is not expressed in the DG (Tanaka et al., 2012). 5-HT1A receptors are also expressed in the CA1 pyramidal cell layer and the CA3 pyramidal cell layer, as well as the granule cell layer, hilus, and SGZ of the DG (Tanaka et al., 2012). 5-HT2A receptors are expressed in the hilus of the DG and the pyramidal cell layer of CA3 (Tanaka et al., 2012). 5-HT3 receptors are expressed in the hilus and SGZ of the DG (Tanaka et al., 2012), whereas 5-HT4 receptors are expressed in the granule cell layer, the SGZ of the DG, and the pyramidal cell layer of the CA1-3 regions (Imoto et al., 2015; Manuel-Apolinar et al., 2005; Tanaka et al., 2012; Vilaró et al., 2005). 5-HT6 receptors are distributed in the CA1-3 and DG regions (Roberts et al., 2002). Moreover, 5-HT2C receptors are expressed only in the CA3 pyramidal cell layer (Tanaka et al., 2012), implicating indirect effects on neurogenic processes.

In the cell proliferation phase (as shown in Fig. 2), neurogenic cells such as Type-1, 2A/B, and 3 cells are under bidirected regulation by multiple 5-HT receptors (Klempin et al., 2010). 5-HT1A receptors facilitate cell proliferation (Banasr et al., 2004; Bert et al., 2006; Günther et al., 2011; Klempin et al., 2010; Niedernhofer et al., 2006; Soumier et al., 2010). Selective stimulation of 5-HT1A receptors increases the number of proliferating cells (Banasr et al., 2004; Bert et al., 2006; Günther et al., 2011; Klempin et al., 2010; Noto et al., 2016; Soumier et al., 2010), while inhibition of 5-HT1A receptors decreases it (Radley and Jacobs, 2002). In these studies, however, the proliferating cells were detected by BrdU labeling; thus, the cell type specificities were not analyzed. Moreover, stimulation of 5-HT3 receptors also facilitates cell proliferation from Type-2B to Type-3 cells, as shown by the double staining of BrdU and DCX (Kondo et al., 2018). The 5-HT4 receptor is also implicated in cell proliferation from Type-1 to Type-3 cells (Imoto et al., 2015; Lucas et al., 2007). Across these proliferation phases, 5-HT2C receptors are inhibitory to it, as investigated by BrdU labeling (Banasr et al., 2004; Klempin et al., 2010).

In the subsequent phases of neurogenesis (i.e., differentiation and maturation), 5-HT signals activate the BDNF pathway (Duman et al., 2001; Mattson et al., 2004). BDNF is released from mature neurons in a normal physiological state (Yohn et al., 2017) and from microglia in response to the administration of 5-HT-related antidepressants (Turkin et al., 2021). The efficacy of BDNF enhancement in pyramidal cells of the CA1-4 regions following chronic antidepressant treatment is evident (Xu et al., 2003). 5-HT2A receptors may inhibit BDNF expression in the DG (Vaidya et al., 1997). The neuronal BDNF signals are transferred to astrocytic cells and stimulate the TrkB pathway (Stahlberg et al., 2018) to be redirected to calretinin-positive neurons (i.e., immature neurons) in the DG (Chan et al., 2008). Accordingly, dendritic BDNF acts to promote neuronal maturation of adult-born neurons in attributes like dendritic length, branching, and granule neuron density (Wang et al., 2015; Waterhouse et al., 2012). There is a direct pathway from 5-HT1A receptors to BDNF synthesis (Ivy et al., 2003). A lack of 5-HT1A receptors in the DG results in the diminished expression of BDNF following chronic SSRI treatments (Samuels et al., 2015). 5-HT signals activate cAMP response element-binding protein (CREB) (Conti et al., 2002; Nibuya et al., 1996), which promotes the transcription of the BDNF gene (Dremencov et al., 2003; Mattson et al., 2004; Schloss and Henn, 2004).

All localized 5-HT receptors, except for the 5-HT3 receptor, are involved in the cAMP-CREB signaling cascade (Marin et al., 2020), indicating that BDNF signals may also be involved in other 5-HT receptor cascades (Mattson et al., 2004). For example, a 5-HT2A/2C receptor agonist (dimethoxyphenylisopropylamine) decreases BDNF mRNA expression in the hippocampus (Vaidya et al., 1997), while a selective 5-HT2C receptor antagonist (S32006) increases it in the DG (Dekeyne et al., 2008), and a 5-HT6 receptor agonist (LY-586713) increases BDNF mRNA in the hippocampus (de Foubert et al., 2007, 2013). In addition, the efficacy of SSRI treatment-facilitating neurogenesis in immature neurons was eliminated in 5-HT4 receptor KO mice (Imoto et al., 2015), suggesting several possible mediatory mechanisms underlying the effect of 5-HT4 receptors on neurogenesis in granule cells. In the hippocampus, the processes of neurogenesis occur in restricted areas, such as the DG and CA3, while several subtypes of 5-HT receptors act to switch entire cell processes of neurogenesis. While cell proliferation is facilitated via 5-HT1A but inhibited via 5-HT2C receptors, the BDNF pathway mediates cell-type differentiation and maturation via the complex of regulation by 5-HT1A, 2C, and 6 receptors.

### 3.3 5-HT-mediated neurogenic processes in the SVZ

The SVZ contains multiple cell populations associated with neurogenic processes, including astrocyte-like neural stem cells (Type-B cells), transit-amplifying precursor cells (Type-C cells), and neuroblasts (Type-A cells). The neurogenic cells derived from the SVZ move into the OB, changing the cell types from Type-C to Type-A cells, and eventually transform into interneurons in the OB (Bressan and Saghatelyan, 2020; Lledo et al., 2008; Lledo and Saghatelyan, 2005). Recent studies suggest that neurogenic cells developed and migrated from the SVZ may also act for neural circuit tuning to adjust several innate behaviors, including male mating in rats (Lau et al., 2011), mate preference in female mice (Mak et al., 2007), and olfactory scent/pheromone discrimination (Bragado Alonso et al., 2019), although these behaviors are olfactory-driven. Accordingly, NPC-derived cell migration from the SVZ can be observed in the striatum (Arvidsson et al., 2002; Dayer et al., 2005) and the cortex (Kreuzberg et al., 2010).

5-HT signals are also involved in the regulation of neurogenesis in the SVZ, wherein the 5-HT neurons from the DRN send axonal projections directly to the SVZ, which is a repository for several 5HT receptors, including 5-HT1A, 5-HT1B/1D, 5-HT2A/2C, and 5-HT3 subtypes (Chen et al., 2012; Hitoshi et al., 2007; Inta et al., 2008; Morales and Bloom, 1997). 5-HT1A receptors promote cell proliferation in the SVZ, consistent with their function in the hippocampus (Banasr et al., 2004; Grabiec et al., 2009; Soumier et al., 2010). 5-HT2C receptors that exhibit an inhibitory effect on hippocampal cell proliferation (Klempin et al., 2010) enhanced cell proliferation in the SVZ, as indicated by an increase in BrdU-labeled cells after an acute systemic injection of a 5-HT2C agonist (RO600175) (Banasr et al., 2004). Moreover, the function of 5HT1B/1D receptors on neurogenic processes is region specific; the modulation of these receptors has little impact on hippocampal cell proliferation, whereas their stimulation by systemic injection of an agonist (sumatriptan) suppresses the cell proliferation process in the SVZ, and the injection of an antagonist (GR127935) facilitates proliferation in the SVZ (Banasr et al., 2004). This suggests that the inhibitory pathway of 5-HT in the SVZ may switch to 5-HT1B/1D from 5-HT2C, which is starkly different from the 5-HT function in the hippocampus.

Neurogenic cell migration from the SVZ occurs when NPCs thrive into the phase of cell differentiation and maturation as Type-A cells (García-González et al., 2017) (Fig. 2B). 5-HT3A receptors are expressed on SVZ-derived Type-A cells when they migrate to the OB and disappear upon cell maturationto become granule cells of the OB (Kreuzberg et al., 2010). Although the exact role of 5HT3A receptors in this cell stage is still unclear, the speed and direction of transportation to the OB may be mediated by 5HT3A receptors (Fomin-Thunemann and Garaschuk, 2022; García-González et al., 2017). The role of 5HT signals in the SVZ in regulating the processes of differentiation and maturation lacks sufficient evidence, which requires further investigation.

### 3.4 5-HT-mediated neurogenic processes in non-NSC-located regions

5-HT neurons widely innervate the cortical and forebrain areas, including the striatum, amygdala, thalamic habenula, and hypothalamus (Hensler, 2012) (Fig. 1C). An increasing amount of evidence suggests that the antidepressant or pharmacological agent-stimulated 5-HT release also induces neurogenesis-like cell activities in these non-NSC-located brain regions (Feliciano et al., 2015). Thus, 5-HT mediation of neurogenic cell activities is not required for the localization of NSCs (Kodama et al., 2004; Ohira, 2022; Sachs and Caron, 2014; Soumier et al., 2010).

Similar to the cell proliferation in the DG and SVZ, cells incorporated with injected BrdU have been found in the striatum and prefrontal cortex (Dayer et al., 2005; Soumier et al., 2010; Stopczynski et al., 2008) (Fig. 2C). The stimulation of 5-HT1A receptors in these regions increases the number of BrdU-labeled cells in these locations (Soumier et al., 2010). Interestingly, 5-HT1A receptors expressed in these regions may have a promoting effect on neurogenic processes, whereas 5-HT2C receptors mediate neurogenic processes in a region-dependent manner (Soumier et al., 2010). Accordingly, cell proliferation-like reactions by BrdU labeling following 5-HT stimulation are observed in the hypothalamus and thalamic habenula, where 5-HT2C receptors predominate (Bombardi et al., 2021; Sachs and Caron, 2014; van Lingen et al., 2019). Chronic treatment with SSRIs increased the numbers of nestin-positive cells in these two regions (Sachs and Caron, 2014), indicating the existence of NSC-like cells (e.g., Type-1, 2A, and 2B cells in the hippocampal DG (Filippov et al., 2003; Kronenberg et al., 2003; Nicola et al., 2015) and Type B and C cells in the SVZ (Doetsch et al., 1997, 1999; Kim et al., 2009; Liu and Crews, 2017; Mirzadeh et al., 2008)).

There are two possible sources of BrdU-labeled neuronal cells in these regions: 1) the presence of NSC-like cells (Duan et al., 2015; Zhang et al., 2017) or 2) the migration of progenitor cells from the SVZ (Chmielnicki et al., 2004; Grade et al., 2013; Jin et al., 2003). Although limited investigations have been performed on these BrdU-labeled cells, a study demonstrated 5-HT mediation in the fate determination of these BrdU-labeled cells in the adult mouse hypothalamus (Ohira, 2022). SSRI treatment increases the ratio of BrdU-labeled neuronal cells to total cells, including astrocytes (Ohira, 2022). However, the majority of BrdU-labeled cells in the striatum and neocortex were positive for the oligodendrocyte precursor, NG2, indicating that these BrdU-labeled cells may not follow neuronal fate but rather glial fate (Dayer et al., 2005). In this context, some studies implicated differences in the cellular features of BrdU-labeled cells in non-NSC-located sites (Feliciano et al., 2015; Tamura et al., 2007). While DCX-positive cells in the NSC-located regions are neuronal (Steiner et al., 2006), the DCX-expressing cells in the neocortex of adult rats continue to be multipotent to become neurons or oligodendrocytes (Tamura et al., 2007). We must elucidate the characteristics of these neurogenic cells in non-NSC sites through different perspectives and strategies, using techniques such as multiple immunostaining or single-cell gene expression analysis (Duan et al., 2015).

## 4 Future remarks

Central 5-HT contributes to their antidepressant effects as the higher extracellular level of 5-HT induced by chronic SSRI treatment enhances neurogenic processes. Several studies investigating the function of SSRIs in neurogenesis in rodents reported that chronic administration of SSRIs enhances the proliferation of NSCs and cell-type differentiation, increases the survival of adult-born neurons, and accelerates the maturation of immature neurons (Malberg et al., 2000). Once 5-HT neurons are activated, increased extracellular levels of 5-HT stimulate different subtypes of 5-HT receptors that are widely expressed in several brain regions. The cell proliferation of neurogenesis is promoted via 5-HT1A receptors consistently across different brain regions, while 5-HT2C receptors have bidirectional effects on neurogenesis in a region-specific fashion. 5-HT receptors also mediate the subsequent phases of neurogenesis, i.e., differentiation and maturation, via the BDNF stimulation pathway. These 5-HT mediations on neurogenic processes are evident not only in two limited regions containing NSCs, the hippocampal DG and the SVZ, but also in brain regions not containing NSCs, such as the cortex, striatum, and hypothalamus.

Investigation of therapeutic pathways to manipulate the brain-wide neurogenic orchestration stimulated by 5-HT in the depressed state, is warranted. Based on the present literature review, we argue that hypothesizing a specific brain region to be responsible for the mediation of depression may not be feasible. It is more likely that 5-HT-mediated neurogenic processes occur in broad areas, and with the aid of neurotrophic factors, neuronal reconstruction and integration are produced. Experimental clarification of these 5-HT circuit-based mediatory mechanisms in the processes of neurogenesis and beyond is fundamental for a better understanding of antidepressant effects through neurogenesis to depression-like states and thus for the further establishment of an antidepressant therapeutic strategy using the intervention of 5-HT signaling.

## Role of the funding source

This work was supported by the Grant-in-Aid for Early-Career Scientists (20K16232 and 23K14359 (YH), 10.13039/501100001691Japan Society for the Promotion of Science) and the Fund for the Promotion of Joint International Research (19K24681 (HA), 10.13039/501100001691Japan Society for the Promotion of Science).

## 5 Disclosure instructions

No generative AI and AI-assisted technologies were used during the preparation of this manuscript.

## Declaration of competing interest

The authors declare that they have no known competing financial interests or personal relationships that could have appeared to influence the work reported in this paper.

## Data Availability

No data was used for the research described in the article.
